# Impact of maintenance immunosuppression regimen on anti-SARS-CoV-2 antibody and cellular kinetics in kidney transplant recipients receiving ChAdOx1 as primary vaccination

**DOI:** 10.1016/j.bjid.2025.104604

**Published:** 2025-12-24

**Authors:** Julia Soares Reis, Roberto Matias Souza, Helio Tedesco-Silva, Lúcio Requião-Moura, José Medina Pestana, Renato Demarchi Foresto

**Affiliations:** a Universidade Federal de São Paulo, Nephrology Division, São Paulo, SP, Brazil; b Fundação Oswaldo Ramos, Hospital do Rim, São Paulo, SP, Brazil

**Keywords:** COVID-19, SARS-CoV-2, Vaccine, Kidney transplant, Immunosuppression

## Abstract

**Background:**

Kidney Transplant Recipients (KTRs) face increased COVID-19 risks due to immunosuppression, which may affect vaccine response. This study evaluates the impact of Mycophenolate Sodium (MPS) versus Azathioprine (AZA) on anti-SARS-CoV-2 humoral and cellular kinetics after ChAdOx1 vaccination.

**Methods:**

In this prospective, observational study, 89 KTRs who seroconverted post-vaccination were grouped based on maintenance immunosuppression (MPS: *n* = 51; AZA: *n* = 38). Anti-SARS-CoV-2 IgG titers, neutralizing antibody activity, and cellular immunity assessed by the Interferon-Gamma (IFN-γ) release were measured at screening and 1-, 3-, 6-, and 12-months post-transplant. Linear regression and generalized estimating equations assessed group and time effects.

**Results:**

At baseline, IgG titers were 12,059.2 AU/mL (MPS) and 14,369.3 AU/mL (AZA), with both groups experiencing a decline at month 1 (9483.9 AU/mL and 11,023.5 AU/mL, respectively). By month 12, titers stabilized at 11,626.8 AU/mL (MPS) and 13,851.4 AU/mL (AZA; *p* = 0.286). Neutralizing activity was initially higher with AZA (0.924 vs. 0.764 at baseline; *p* = 0.006) but converged by month 3 (0.937 vs. 0.871; *p* = 0.161). Booster doses significantly enhanced neutralizing activity by 0.175 over 12 months. Positive cellular immunity was inferior in the MPS group at screening (4.2% vs. 15.8 %; *p* = 0.171) and M1 (13.3% vs. 27.8 %; *p* = 0.266), but similar in the following visits.

**Conclusions:**

AZA provided a transient early advantage in immunity responses against SARS-CoV-2, but long-term humoral and cellular kinetics were comparable. Booster doses are essential for sustaining immunity in KTRs, emphasizing the need for tailored vaccination strategies. These findings may inform clinical decisions during pandemics.

## Introduction

The COVID-19 pandemic has posed a significant global health challenge, particularly for vulnerable populations such as Kidney Transplant Recipients (KTRs).^1^ Due to the chronic use of immunosuppressive drugs to prevent graft rejection, these patients face a heightened risk of severe complications and mortality from SARS-CoV-2 infection.^1^ Moreover, their immune response to vaccination may be impaired, raising concerns about the effectiveness of COVID-19 vaccines in this population.^2^

The ChAdOx1 (AstraZeneca) is a viral vector-based vaccine in which an adenovirus that infects chimpanzees is genetically engineered to insert the SARS-CoV-2 Spike protein. The primary schedule comprises two intramuscular shots administered at 4 to 12 weeks, with efficacy against infection above 70 % in the general population.^3^^,^^4^ In KTRs, the ChAdOx1 vaccine provides comparable antibody titers to mRNA platforms.^5^ Notably, while vaccination has not significantly reduced the incidence of COVID-19 in KTRs, an increased number of vaccine doses has been associated with a decreased risk of mortality in this population.^6^

In addition to the lower humoral response, the duration of kinetics of anti-SARS-CoV-2 antibodies appears to be disadvantageous in those using chronic immunosuppression. However, the association between the type of maintenance immunosuppressive therapy and the kinetics of the humoral and cellular responses after transplantation is not well established. In this context, it becomes crucial to investigate whether the type of maintenance immunosuppression influences the kinetics of anti-SARS-CoV-2 antibodies and Interferon-Gamma (IFN-γ) release in kidney transplant recipients who were primarily vaccinated with ChAdOx1 and had a primary response.

## Methods

### Study design and population

This is a prospective, observational, single-center study comparing the kinetics of the anti-SARS-CoV-2 antibodies and Interferon-Gamma (IFN-γ) release in patients receiving a kidney transplant who received the ChAdOx1 vaccine as the primary vaccine schedule according to the maintenance immunosuppressive regimen prescribed on the first day after transplantation.

This is a post-hoc analysis of a prospective study previously published comparing the kinetics of SARS-CoV-2 immune response in dialysis patients and those undergoing a kidney transplant.^7^ For the primary study, the investigators calculated a sample size of 114 individuals to be included in each group. For this post hoc analysis, we selected those (*n* = 89) who received the ChAdOx1 nCoV-19 as primary schedule for vaccination, aiming to review the effect of the type of maintenance immunosuppression on humoral and cellular kinetics.

We included patients who received the ChAdOx1 vaccine as the primary schedule and had undergone kidney transplantation in our center from a living or deceased donor. We excluded patients with a previous diagnosis of COVID-19, negative SARS-CoV-2 IgG serology on inclusion, those living with HIV, and those currently undergoing cancer treatment. All patients were followed up for 12-months from the date of inclusion. A blood sample was collected immediately before the transplant and after 1-, 3-, 6-, and 12-months for immunological analysis. In each study visit, the patients were argued about vaccine boosters received and COVID-19 diagnosis.

The eligible population signed an informed consent form. The study was complied with the Good Clinical Practice guidelines and the Declaration of Helsinki and was approved by the Institutional Review Board of the Federal University of São Paulo (approval number: 5.056.135).

### Study objectives

The main objective of the study was to compare the kinetics of anti-SARS-CoV-2 IgG titers and neutralizing antibodies, and the Interferon-Gamma (IFN-γ) release in de novo kidney transplant recipients receiving mycophenolate or azathioprine as part of the immunosuppressive regimen over a 12-month follow-up period. Secondary assessments were conducted at 1-, 3-, and 6-months.

Other objectives were to assess the number, type, and time of additional vaccine doses, relevant clinical outcomes such as COVID-19 incidence, death, all-cause hospitalization, and transplant outcomes such as cytomegalovirus infection, delayed graft function, acute rejection, and graft loss. These outcomes were also analyzed and compared between the groups at 1-, 3-, 6-, and 12-months.

### Immunological assessment

The humoral immune was assessed by the kinetics of the SARS-CoV-2, IgG titers, and neutralizing antibodies in blood samples collected at five study visits: screening (immediately before the transplant), and study visits after 1-, 3-, 6-, and 12-months of inclusion.

We used the AdviseDx SARS-CoV-2 IgG II test (Abbott Laboratories, Sligo, Ireland; lower limit of positivity 50 AU/mL) to evaluate IgG antibodies targeting the receptor-binding domain (RBD) of the S1 subunit of the SARS-CoV-2 Spike protein.^8^ We used the cPass™ SARS-CoV-2 test (GenScript Laboratory, Rijswijk, The Netherlands; positivity limit 30 %) to evaluate the neutralizing activity of anti-SARS-CoV-2 antibodies.^9^

Cell-mediated immunity was assessed using the QuantiFERON SARS-CoV-2 RUO Starter Set (QIAGEN), which measures Interferon-Gamma (IFN-γ) release in response to SARS-CoV-2-specific antigens. Whole blood was collected into three tubes (Nil, Ag3, and Mitogen), incubated, and processed according to the manufacturer’s instructions. Results were interpreted as positive, negative, or indeterminate.^10^

### Vaccination strategy

In our country, the COVID-19 vaccination was managed by the National Immunization Program, and the public health system applied all vaccines. The KTRs have been prioritized for receiving vaccine doses and boosters since May 2021. During this study, all patients received at least one vaccine booster by the public health system. Our study did not intervene on vaccination policies of these patients.

The ChAdOx1 nCoV-19 is a non-human adenovirus vector vaccine given in two doses, with an interval of four to twelve weeks, developed by AstraZeneca.^11^ The study used to register the vaccine included 32,451 adult volunteers to receive the ChAdOx1 nCoV-19 vaccine or placebo, in a 2:1 ratio. The efficacy in preventing infection was 74 %, and 83.5 % in people over 65-years of age.^11^

### Immunosuppressive regimen

According to the standard institutional protocol, all kidney transplant recipients undergo intravenous induction therapy, which includes 1 g of methylprednisolone administered intraoperatively and a single dose of rabbit Anti-Thymocyte Globulin (rATG) at 3 mg/kg on the first postoperative day. This study did not involve any interventions or modifications to the established immunosuppressive regimen. Maintenance therapy comprises prednisone 0.5 mg/kg QD tapered to 5 mg QD until the end of the first month and tacrolimus 0.05‒0.1 mg/kg BID for all patients. The third drug consists of mycophenolate sodium 720 mg BID or azathioprine 2 mg/kg QD, selected based on donor characteristics (standard or expanded criteria donor) and the recipient's immunological risk profile.

### Statistical analysis

Categorical variables were expressed as percentages and compared using the Chi-Square test. Numerical variables were evaluated for normality using the Kolmogorov-Smirnov test. Quantitative variables were presented as a median and interquartile range for non-parametric variables and as a mean and standard deviation for parametric variables. Differences between groups were analyzed using the Mann-Whitney test.

We performed a linear regression using Generalized Estimated Equations (GEE) with pairwise comparison adjusted by Bonferroni to compare the time and group effect on anti-SARS-CoV IgG titers and neutralizing antibodies over time.^12^ Due to the small number of negative and indeterminate results, neutralizing antibody data were dichotomized into positive and non-positive.

Statistical analyses were performed using the statistical package SPSS version 29 (IBM Corp. Released 2022. IBM SPSS Statistics for Windows, Version 29.0, Armonk, NY, USA: IBM Corp.). We considered a *p* < 0.05 as statistically significant.

## Results

### Demographic characteristics

**Fig. 1:**
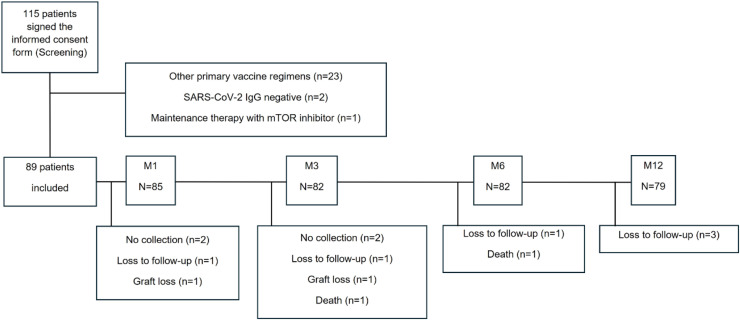
Flowchart of the study population.

Between December 13, 2021 and April 05, 2022, 115 patients were enrolled, but 26 were excluded due to other primary vaccine regimens (*n* = 23), SARS-CoV-2 IgG negative at screening (*n* = 2), or receiving mTOR inhibitor as maintenance immunosuppressive therapy (*n* = 1), resulting in 89 patients included in this study (Fig. 1). They all had a blood sample collected at screening and a positive anti-SARS-CoV-2 IgG serology.

The median age was 47.1-years, most were male (52.8 %) and white (54 %). The most common etiology of Chronic Kidney Disease (CKD) was undetermined (30 %), followed by chronic glomerulonephritis (27 %), diabetes mellitus (21.3 %), autosomal dominant polycystic kidney disease (7.0 %), urological diseases (5.6 %) and hypertension (4.5 %).

Comorbidities such as hypertension and diabetes mellitus were present in 80.9 % and 22.5 % of patients, respectively. Hemodialysis was the most common therapy of CKD (83.1 %), with median duration of 29.2-months. Of 89 patients, 51 received mycophenolate sodium and 38 received azathioprine as maintenance immunosuppressive regimen. In both groups, the median HLA mismatches in loci A, B and DR was 2, and panel-reactive antibody was 27.5 % and 7.9 %, respectively. Of all, 2.2 % of patients received a retransplantation (Table 1).

**Table 1:** Demographic characteristics of the study population.

	Total (n = 89)	Mycophenolate (n = 51)	Azathioprine (n = 38)	p
KTRs				
Age, years (IQR)	47.1 (35.8‒58.8)	50.4 (35.9‒58.9)	44.3 (35.7‒57.0)	0.545
Male, n ( %)	47 (52.8)	21 (41.2)	26 (68.4)	0.011
Ethinicity, n ( %)				0.053
White	54 (60.7)	28 (54.9)	26 (68.4)	
Mixed	28 (31.5)	16 (31.4)	12 (31.6)	
Black	7 (7.9)	7 (13.7)	0 (0.0)	
Hypertension, n ( %)	72 (80.9)	38 (74.5)	34 (89.5)	0.076
Diabetes mellitus, n ( %)	20 (22.5)	13 (25.5)	7 (18.4)	0.429
Cause of CKD, n ( %)				0.307
Undetermined	30 (33.7)	12 (23.5)	18 (47.4)	
Glomerulonephrites	24 (27.0)	15 (29.4)	9 (23.7)	
Diabetes Mellitus	19 (21.3)	13 (25.5)	6 (15.8)	
Polycystic Kidney Disease	7 (7.9)	5 (9.8)	2 (5.3)	
Urological	5 (5.6)	3 (5.9)	2 (5.3)	
Hypertension	4 (4.5)	3 (5.9)	1 (2.6)	
Time on dialysis, months (IQR)	29.2 (16.2‒54.4)	33.3 (23.3‒61.6)	21.0 (11.8‒43.8)	0.006
Type of dialysis, n ( %)				0.068
Hemodialysis	74 (83.1)	46 (90.2)	28 (73.7)	
Peritoneal dialysis	9 (10.1)	4 (7.8)	5 (13.2)	
Preemptive	6 (6.7)	1 (2.0)	5 (13.2)	
HLA ABDR Mismatch, n (IQR)	2 (1‒3)	2 (1‒3)	2 (0‒3)	0.100
PRA > 0 %, n ( %)	17 (19.1)	14 (27.5)	3 (7.9)	0.020
Retransplantation, n ( %)	2 (2.2)	1 (2.0)	1 (2.6)	0.833

KTR, Kidney Transplant Recipient; n, Number; IQR, Interquartile Range; CKD, Chronic Kidney Disease; HLA, Human Leukocyte Antigen; PRA, Panel-Reactive Antibody.

The median donor age was 50-years, 48 % were male and 50 % were white. Kidneys from a deceased donor were transplanted in 76 % of the patients; 30 % expanded criteria donors, with a median KDPI of 55 %, final creatinine of 1.41 mg/dL, and cold ischemia time of 22.8 h. In the MPS group, donors were older (54.0 vs. 38.0 years; *p* < 0.001) and filled the expanded criteria in 98.0 % vs. 68.4 % (*p* < 0.001). The proportion of male donors (60.8 % vs. 44.7 %; *p* = 0.133) and white donors (54.9 % vs. 57.9 %; *p* = 0.761) were similar between the groups.

### Vaccination status at screening and boosters received during the study

As this study was conducted during the COVID-19 pandemic and transplant patients were prioritized for boosters, most patients (*n* = 69; 77.5 %) had already received at least one booster at inclusion (Table 2, Table 3). Of these, 63 received three doses, and 6 patients received four doses, with no difference in the distribution between groups. The time elapsed from the last dose of vaccine administered to inclusion in the study was the same for both groups (90-days; *p* = 0.990), with the BNT162b2 vaccine being preferentially administered in both groups (51 % and 65.8 %) (Table 2, Table 3). About the other vaccines, 23.6 % of patients received ChAdOx1 nCoV-19, 18 % received CoronaVac and 1.1 % Ad26.COV2.S (Table 2, Table 3). Among the patients who received the third and fourth doses of vaccine before the inclusion, the BNT162b2 vaccine was the preferred in both groups (67.6 % and 82.8 %; *p* = 0.273) and (60 % and 100 %; *p* = 0.741) (Table 3).

**Table 2:** Vaccination status of the study population at screening.

	Total (n = 89)	Mycophenolate (n = 51)	Azathioprine (n = 38)	p
Doses received, n ( %)				0.367
2	20 (22.5)	12 (23.5)	8 (21.1)	
3	63 (70.8)	34 (66.7)	29 (76.3)	
4	6 (6.7)	5 (9.8)	1 (2.6)	
Last dose of vaccine, n ( %)				0.305
BNT162b2	51 (57.3)	26 (51.0)	25 (65.8)	
ChAdOx1 nCoV-19	21 (23.6)	12 (23.5)	9 (23.7)	
CoronaVac	16 (18.0)	12 (23.5)	4 (10.5)	
Ad26.COV2.S	1 (1.1)	1 (2.0)	0 (0.0)	
Time since last dose, days (IQR)	90 (56‒133)	90 (56‒126)	90 (54.7‒133)	0.990

n, Number; IQR, Interquartile Range.

**Table 3:** Type of vaccine received by patients at screening.

3 doses received, n ( %)	Total (n = 63)	Mycophenolate (n = 34)	Azathioprine (n = 29)	p
3rd dose				0.141
BNT162b2	47 (74.6)	23 (67.6)	24 (82.8)	
CoronaVac	15 (23.8)	11 (32.4)	4 (13.8)	
ChAdOx1 nCoV-19	1 (1.6)	0 (0.0)	1 (3.4)	
4 doses received, n ( %)	Total (n = 6)	Mycophenolate (n = 5)	Azathioprine (n = 1)	p
3rd dose				0.273
BNT162b2	3 (50.0)	3 (60.0)	0 (0.0)	
CoronaVac	3 (50.0)	2 (40.0)	1 (100.0)	
4th dose				0.741
BNT162b2	4 (66.7)	3 (60.0)	1 (100.0)	
CoronaVac	1 (16.7)	1 (20.0)	0 (0.0)	
ChAdOx1 nCoV-19	1 (16.7)	1 (20.0)	0 (0.0)	

n, Number.

**Fig. 2:**
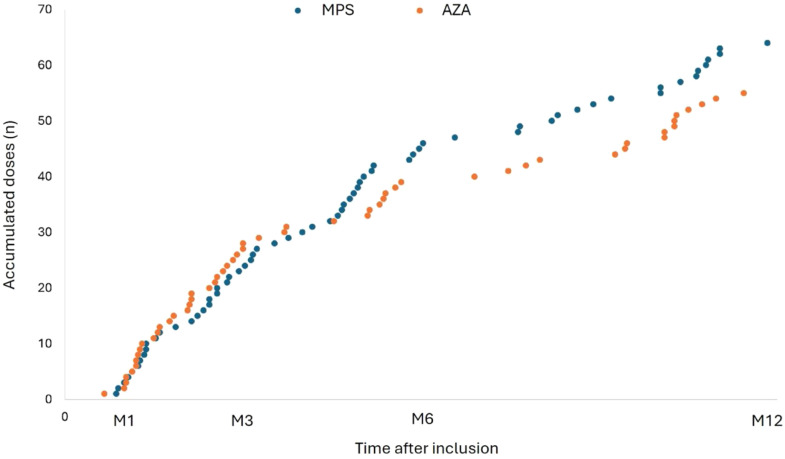
Time of booster and number of accumulated doses in both groups after inclusion. Each point represents the accumulated number of additional vaccine doses in patients receiving mycophenolate (blue) or azathioprine (orange).

This study followed the national immunization policy against COVID-19 during the pandemic, and patients continued to receive boosters during the study follow-up and the vaccines available then. During the 12-month follow-up period, the study patients received 114 additional vaccine doses, 61 in the MPS group and 53 in the AZA group (*p* = 0.195). The distribution of boosters was very similar between the groups (Fig. 2).

Most of the boosters were received until M3, with the AZA group receiving the most doses in this period (62.2 % vs. 37.8 %; *p* = 0.028). At M6, the MPS group received more doses than the AZA group (46.7 % vs. 27 %; *p* = 0.068). In addition, the vaccine of choice for both groups during the study was BNT162b2 (Table 4). The time between the last dose and the study visit was similar in both groups, except at M3, with 102 vs. 55 days in the MPS and AZA groups, respectively (*p* = 0.007) (Table 4).

**Table 4:** Boosters received, type of vaccine and time since last dose.

	M1			M3		
	MPS (n = 49)	AZA (n = 36)	p	MPS (n = 45)	AZA (n = 37)	p
Booster, n ( %)	3 (6.1)	4 (11.1)	0.408	17 (37.8)	23 (62.2)	0.028
Vaccine, n ( %)			0.268			0.015
BNT162b2	3 (100)	1 (25)		10 (58.8)	13 (56.5)	
ChAdOx1 CoV-19	0 (0)	1 (25)		4 (23.5)	0 (0)	
Ad26.COV2.S	0 (0)	1 (25)		1 (5.9)	9 (39.1)	
CoronaVac	0 (0)	1 (25)		2 (11.8)	1 (4.3)	
Last dose^a^, days (IIQ)	121 (82‒148)	107 (68‒166)	0.513	102 (49‒183)	55 (25‒101)	0.007
	M6		M12			
	MPS (n = 45)	AZA (n = 37)	p	MPS (n = 44)	AZA (n = 35)	p
Booster, n ( %)	21 (46.7)	10 (27)	0.068	20 (45.5)	16 (45.7)	0.982
Vaccine, n ( %)			0.979			0.717
BNT162b2	11 (52.4)	5 (50)		14 (70)	10 (62.5)	
ChAdOx1 CoV-19	5 (23.8)	2 (20)		1 (5)	1 (6.3)	
Ad26.COV2.S	3 (14.3)	2 (20)		0 (0)	1 (6.3)	
CoronaVac	2 (9.5)	1 (10)		5 (25)	4 (25)	
Last dose^a^, days (IIQ)	108 (62‒146)	108 (77‒140)	0.744	185 (89‒266)	179 (66‒288)	0.933

M, Month; n, Number; IQR, Interquartile Range.

a Time between the last booster and the study visit.

### SARS-CoV-2 IgG kinetics

**Fig. 3:**
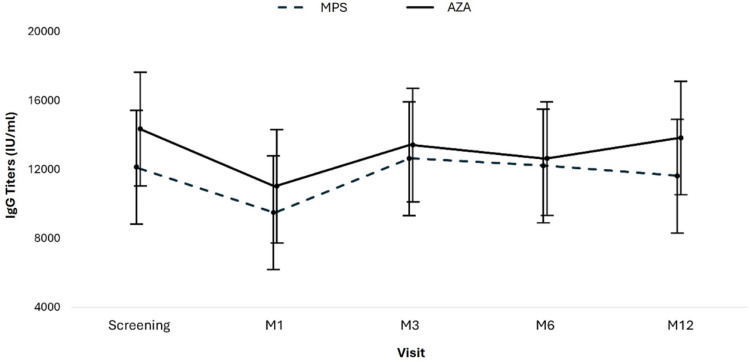
Evolution of anti-SARS-CoV-2 IgG antibody titers. Means and 95 % CI for anti-SARS-CoV-2 IgG antibody titers by group and study visit. Test used: AdviseDx SARS-CoV-2 IgG II test, Abbott Laboratories; lower limit of positivity 50 AU/mL.

Anti-SARS-CoV-2 IgG antibody titers were assessed at screening and in months-1, −3, −6, and −12 and compared according to the groups. As summarized in Table 5 and Fig. 3, IgG titers in the AZA group were numerically but not statistically superior compared to patients receiving MPS at all study visits (*p* = 0.286). In both groups, IgG titers varied similarly over time.

**Table 5:** Mean and confidence interval of anti-SARS-CoV-2 IgG antibody titers at each study visit.

	Total (n = 89)	MPS (n = 51)	AZA (n = 38)	p
Visits, Mean (95 % CI)				0.286
Screening	13,214.3 (10,442.3 – 15,986.3)	12,059.2 (8307.4 – 15,811.0)	14,369.3 (10,287.7 – 18,450.9)	
M1	10,253.7 (7647.5 – 12,859.9)	9483.9 (5868.8 – 13,099.1)	11,023.5 (7268.4 – 14,778.5)	
M3	13,046.1 (10,181.4 – 15,910.9)	12,660.0 (8721.7 – 16,598.2)	13,432.3 (9271.0 – 17,593.7)	
M6	12,424.8 (9574.1 – 15,275.4)	12,225.8 (8430.7 – 16,020.9)	12,623.8 (8369.1 – 16,878.4)	
M12	12,739.1 (9723.5 – 15,754.8)	11,626.8 (7850.9 – 15,402.8)	13,851.4 (9148.4 – 18,554.5)	

CI, Confidence Interval; MPS, Mycophenolate Sodium; AZA, Azathioprine; M, Month.

Fig. 3 shows the means and confidence intervals of the anti-SARS-CoV-2 IgG antibody titers by group, by study visit, and the descriptive levels of the linear models with longitudinal random effects. In this model, the effects of three components are evaluated: time, group, and interaction between group and time (Fig. 3).

We performed a linear regression by GEE (Generalized Estimated Equations) with pairwise comparison adjusted by Bonferroni to compare the groups over time. A total of 417 measures were evaluated across all the study visits: 234 in the mycophenolate group and 183 in the azathioprine group. Throughout the study period, the mean antibody titers were similar in the mycophenolate group (11,611.2 IU/mL; 95 % CI: 8878.6‒14,343.7) and the azathioprine group (13,060.1 IU/mL; 95 % CI: 10,237.1–15,883.1) (*p* = 0.470; adjusted by Bonferroni) (Fig. 3; Table 6).

**Table 6:** Multiple linear regression model to evaluate the effect of time and type of immunosuppressive regimen on anti-SARS-CoV-2 IgG titers over 12-months.

	Coefficient (95 % CI)	p
Azathioprine (ref. Mycophenolate)	2310.1 (−3233.9 to 7854.1)	0.414
Visit (ref. Screening)		0.040
M1	−2575.3 (−5812.5 to 661.9)	0.119
M3	600.7 (−4437.8 to 5639.3)	0.815
M6	166.6 (−4820.4 to 5153.6)	0.948
M12	−432.4 (−5892.2 to 5027.4)	0.877
Interaction between group and time (ref. Mycophenolate-Scr)		0.286
Azathioprine – M1	−770.6 (−5991.1 to 4449.9)	0.772
Azathioprine – M3	−1537.7 (−9245.1 to 6169.7)	0.696
Azathioprine – M6	−1912.1 (−9462.9 to 5638.7)	0.620
Azathioprine – M12	−85.5 (−7475.6 to 7304.6)	0.982

CI, Confidence Interval; Ref, Reference; M, Month; Scr, Screening.

Considering the entire study population, time affected the mean antibody titers, mainly due to the drop in the mean antibodies in both groups at visit M1. Antibody titers recovered at visit M3, and the means stabilized at visits M6 and M12 (Fig. 3; Table 6).

Analyzing the effect of the interaction between time and the type of immunosuppressive regimen, there was no difference between the groups (*p* = 0.286; Table 6).

### Neutralizing antibodies

**Fig. 4:**
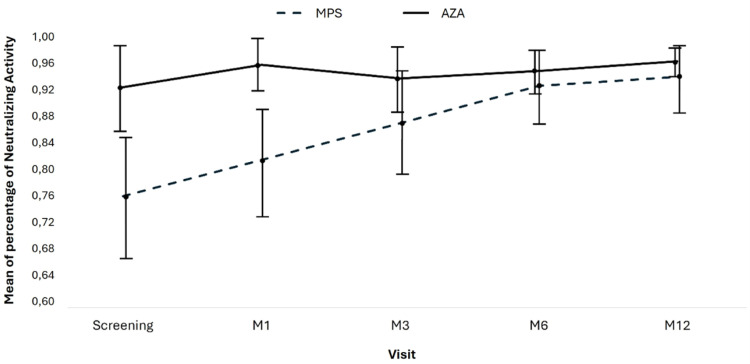
Evolution of neutralizing antibodies. Means and 95 % CI for anti-SARS-CoV-2 IgG neutralizing antibodies by group and study visit. Test used: AdviseDx SARS-CoV-2 IgG II test, Abbott Laboratories; lower limit of positivity 50 AU/mL.

The neutralizing activity of antibodies between MPS and AZA groups at Screening (0.764 vs. 0.924; *p* = 0.006) and M1 (0.817 vs. 0.957; *p* = 0.004) was significantly different, but it was similar in M3, M6, and M12 (Table 7; Fig. 4). In the AZA group, the mean neutralizing activity remained stable over time, but there was a significant increase in the neutralizing activity of patients in the MPS group.

**Table 7:** Mean and confidence interval of anti-SARS-CoV-2 antibody neutralizing activity at each study visit.

	Total (n = 89)	MPS (n = 51)	AZA (n = 38)	p
Visits, Mean (95 % IC)				0.008
Screening	0.844 (0.789 – 0.899)	0.764 (0.671 – 0.857)	0.924 (0.865 – 0.983)	0.006
M1	0.887 (0.841 – 0.932)	0.817 (0.735 – 0.898)	0.957 (0.917 – 0.997)	0.004
M3	0.904 (0.859 – 0.949)	0.871 (0.793 – 0.949)	0.937 (0.892 – 0.983)	0.161
M6	0.937 (0.906 – 0.968)	0.926 (0.873 – 0.979)	0.948 (0.916 – 0.979)	0.502
M12	0.950 (0.925 – 0.976)	0.939 (0.892 – 0.985)	0.962 (0.941 – 0.982)	0.383

CI, Confidence Interval; MPS, Mycophenolate Sodium; AZA, Azathioprine; M, Month.

We performed a linear regression by GEE (Generalized Estimated Equations) with a pairwise comparison to compare the groups over time. A total of 404 measures were evaluated over all the study visits: 224 in the mycophenolate group and 180 in the azathioprine group. Considering all the measurements, on average, the neutralizing activity of the antibodies was higher in the AZA group at 0.082 (95 % CI 0.021 to 0.143; *p* = 0.008, adjusted by Bonferroni).

Considering the entire study period, there was an increase in antibody-neutralizing activity, with an average gain of 0.175 (95 % CI 0.089 to 0.260; *p* = 0.001, adjusted by Bonferroni) between the screening visit and M12.

There was also a group and time effect with a reduction of −0.137 (95 % CI −0.241 to −0.033; *p* = 0.017, adjusted by Bonferroni) reduction in the difference in neutralizing antibody activity at 12-months in the AZA group compared to the MPS group, primarily due to increased neutralizing activity in the AZA group (Table 8).

**Table 8:** Multiple linear regression analysis of time and immunosuppressive regimen effects on mean anti-SARS-CoV-2 neutralizing activity over 12-months.

	Coefficient (95 % CI)	p
Azathioprine (ref. Mycophenolate)	0.082 (0.021 to 0.143)	0.008
Visit (ref. Screening)		0.002
M1	0.053 (−0.01 to 0.115)	0.097
M3	0.107 (0.025 to 0.189)	0.010
M6	0.162 (0.079 to 0.245)	0.001
M12	0.175 (0.089 to 0.260)	0.001
Interaction between group and time (ref. Mycophenolate-Scr)		0.017
Azathioprine – M1	−0.020 (−0.101 to 0.061)	0.634
Azathioprine – M3	−0.094 (−0.203 to 0.015)	0.092
Azathioprine – M6	−0.138 (−0.237 to −0.040)	0.006
Azathioprine – M12	−0.137 (−0.241 to −0.033)	0.010

CI, Confidence Interval; Ref, Reference; M, Month; Scr, Screening.

**Fig. 5:**
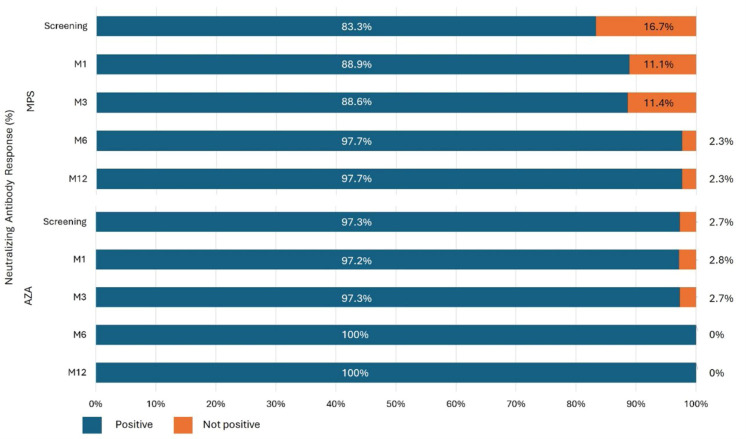
Distribution of neutralizing antibody responses by group and study visit. Incidence of positive and non-positive neutralizing antibodies between groups. M, Month. Test used: cPass™ SARS-CoV-2 test, GenScript Laboratory; positivity threshold 30 %.

Neutralizing antibodies were high in both groups and at all study visits (Fig. 5). At screening, positive neutralizing activity was higher in the AZA group (83.3 % vs. 97.3 %; *p* = 0.038). However, during the follow-up and boosters received by both groups, this difference was attenuated: M1 (88.9 % vs 97.2 %; *p* = 0.155), M3 (88.6 % vs. 97.3 %; *p* = 0.138), M6 (97.7 % vs. 100 %; *p* = 0.351), and M12 (97.7 % vs. 100 %; *p* = 0.383) (Fig. 5).

### Cellular immunity

**Fig. 6:**
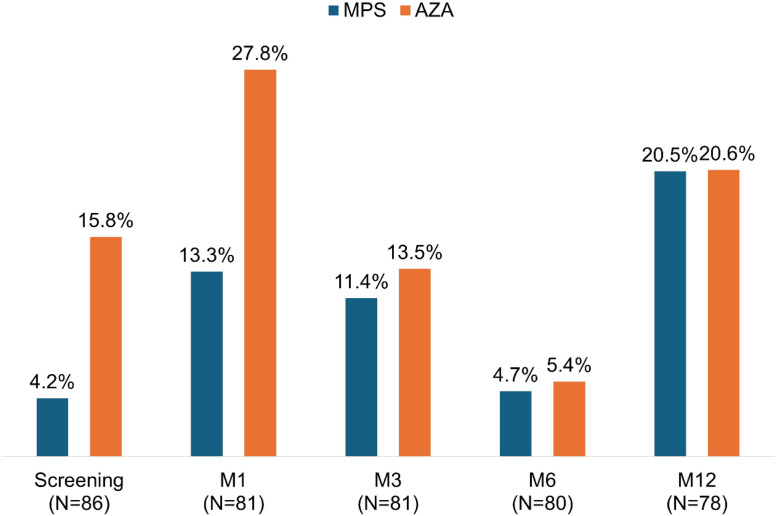
Positive results of QuantiFERON IFN-γ release by group and study visits.

As described on Methods, all patients included in this study had a positive anti-SARS-CoV-2 antibody detected at screening. But, on the day of the transplant, only 4.2 % of patients in the MPS group and 15.8 % in the AZA group had a positive cellular immunity test (*p* = 0.171). After one month, IFN-γ detection increased in both groups, but maintaining the advantage in the AZA group (13.3 % vs. 27.8 %; *p* = 0.266). On the following visits, the detection of IFN-γ release were similar in both groups: M3 (11.4 % vs. 13,5 %; *p* = 0.813), M6 (4.7 % vs. 5.4 %; *p* = 0.779), and M12 (20.5 % vs. 20.6 %; *p* = 0.807) (Fig. 6). Indeterminate results predominated in all study visits in both groups: screening (91.7 % vs. 78.9 %), M1 (68.9 % vs. 58.3 %), M3 (52.3 % vs. 56.8 %), M6 (58.1 % vs. 64.9 %), and M12 (20.5 % vs. 26.5 %).

### Clinical outcomes

During the 12-month follow-up period, we also collected exploratory data on relevant clinical outcomes. We observed 18 cases of COVID-19 in the MPS group and 11 cases in the AZA group (*p* = 0.527), occurring earlier after transplantation in the MPS group (45 vs. 107 days; *p* = 0.003). There were three deaths, all of them in the MPS group – one due to urinary tract infection, and two due to COVID-19, at day 27 and day 50 after transplantation. All-cause hospitalizations also occurred more frequently in the MPS (68.6 % vs. 36.8 %; *p* = 0.003).

Regarding transplant outcomes, the MPS group had a higher incidence of delayed graft function (60.8 % vs. 36.8 %; *p* = 0.025) and cytomegalovirus infection (56.9 % vs. 13.2 %; *p* < 0.001). Acute rejection (7.8 % vs. 7.9 %; *p* = 0.993) and graft loss (9.8 % vs. 2.6 %; *p* = 0.182) were similar between the groups.

## Discussion

This prospective observational study provides an in-depth analysis of the kinetics of anti-SARS-CoV-2 antibody titers, neutralizing activity, and cellular immunity assessed by the Interferon-Gamma (IFN-γ) release in KTRs undergoing maintenance immunosuppression with Mycophenolate Sodium (MPS) or Azathioprine (AZA) after receiving the primary immunization with the ChAdOx1 nCoV-19 vaccine. Our results demonstrated that both MPS and AZA groups exhibited an initial decline in IgG titers at Month 1, followed by stabilization through Month 12. Although IgG titers were numerically higher in the AZA group at all time points, the differences were not statistically significant. Conversely, neutralizing antibody activity was initially higher in the AZA group but converged between the groups by Month 3, remaining comparable thereafter. Our cohort also provided insights into the behavior of cellular responses after vaccination. We observed that IFN-γ detection was infrequent in both groups even in the screening and showed an irregular pattern during the follow-up, with a high proportion of indeterminate results.

The primary response to vaccination and humoral kinetics in KTRs are deficient compared to the general population or dialysis patients and are attributed to the chronic use of immunosuppressive drugs.^13^^,^^14^ The gold standard immunosuppression regimen in kidney transplantation consists of tacrolimus, mycophenolate, and steroids, with few centers having experience in using AZA instead of MPS in patients at low immunological risk, despite similar efficacy and safety in this population.^15^^,^^16^ During the pandemic, several studies indicated MPS as a predictor of mortality and as a predictor of lower vaccine response, but not AZA.^17^^,^^18^ This study was based on the hypothesis that in patients vaccinated against SARS-CoV-2 and seroconverted, the kinetics of humoral and cellular immune response could be different after kidney transplantation according to the immunosuppressive regimen used (MPS vs. AZA).

The decay of antibody titers is a natural process in all individuals and has also been demonstrated for the COVID-19 vaccine. For instance, Sanders et al. compared the decay of anti-SARS-CoV-2 IgG antibody titers between healthy individuals and patients on a spectrum of chronic kidney disease – including KTRs – who received two doses of an mRNA vaccine.^19^ After the vaccination schedule completion, the antibody titers significantly decayed over time in all groups, but KTRs had notably lower antibodies during the 6-month follow-up, suggesting that immunosuppression may play a role in reducing the period of protection offered by the vaccine.^19^ Another study comparing patients on dialysis and KTRs demonstrated that KTRs had lower SARS-CoV-2 IgG titers and neutralizing antibodies over 12-month follow-up, even though KTRs received more boosters during the follow-up.^14^ These results raise the importance of further doses as a primary source of protection for this especially vulnerable population.

Previous studies have demonstrated that cellular immune responses to SARS-CoV-2 vaccination are significantly attenuated in KTRs compared with dialysis patients and immunocompetent controls.^20^ In transplant cohorts, impaired T-cell reactivity has been associated with the immunosuppressive drugs, older age, and IGRA-based assays often yield low or indeterminate results, underscoring a pharmacological role and methodological limitations.^10^^,^^20^ Also, some studies suggest that T-cell responses may precede or even exceed seroconversion in KTRs, indicating that humoral response alone may underestimate protection.^21^ In contrast, dialysis patients generally can generate more robust and sustained cellular responses, with booster doses enhancing both antibody and T-cell immunity, although a gradual decline is still observed over time.^19^^,^^22^^,^^23^ But, even though patients with impaired kidney function can mount measurable spike-specific T-cell responses after vaccination, these responses are usually at lower magnitude compared with healthy controls.^22^^,^^23^

In KTRs, the effectiveness and antibody titers induced by ChAdOx1 were comparable to mRNA vaccines.^5^ For instance, Callaghan et al. demonstrated that two doses of the ChAdOx1 vaccine in KTRs reduced mortality by 20 % compared to those unvaccinated, although the disease incidence was similar.^6^ In Brazil, four vaccines were approved for use during the pandemic: ChAdOx1, BNT162b2, Ad26.COV2.S, and CoronaVac. Because KTRs were at high risk of poor outcomes after COVID-19, they were prioritized early for vaccination, and the majority had already received three or four doses at the time of inclusion. Once our observational study did not modify the healthcare authority recommendations during the pandemic, all individuals received at least one further dose during the study, with the vaccine available at that time, with some accumulating up to six doses.

Ajlan et al. reported that MPS use or triple immunosuppression was associated with a poor humoral response in KTRs vaccinated compared to other regimens containing AZA or mTOR inhibitors.^5^ However, the influence of different maintenance immunosuppressive drugs on the kinetics of humoral and cellular immunity against SARS-CoV-2 in previously vaccinated and seroconverted patients undergoing kidney transplantation has not yet been elucidated. While azathioprine’s mechanism as a purine synthesis inhibitor might offer a more favorable environment for vaccine response than mycophenolate, further studies are needed to clarify the underlying immunological pathways.

Real-world evidence in KTRs indicates that even modest or waning antibody responses may translate into meaningful clinical protection. A large registry data demonstrated that vaccinated solid organ transplant recipients had significantly reduced risks of infection-related hospitalization and death compared to unvaccinated counterparts, confirming that vaccine-induced immunity confers survival benefits despite attenuated responses.^24^ However, because even modest responses decline over time, it becomes essential to characterize the kinetics of this decay in transplant recipients. A clear understanding of the rate and timing of humoral and cellular waning may help optimize booster strategies, ensuring that additional doses are administered before long gaps of immune vulnerability.

The observed kinetics of humoral and cellular immune activity have several implications for clinical practice. First, the initial lag in immune response among KTRs highlights the critical need for timely booster doses to sustain immunity, particularly to emerging SARS-CoV-2 variants. Second, the lack of significant differences in long-term antibody and cellular kinetics between the MPS and AZA groups suggests that vaccine efficacy may be driven primarily by external factors, such as booster doses and the timing of vaccination relative to transplantation. In our study, the type of vaccine received as a booster was similar between groups, as was the time between the last dose and the study visit. This underscores the importance of robust public health strategies to prioritize vaccine access for vulnerable patients, particularly during outbreaks or epidemics. Third, our findings raise questions about immunosuppression manipulation during pandemic scenarios. Given the nonsignificant advantage in humoral and cellular responses observed between the drugs, clinicians may focus on patient-specific prevention strategies instead of reducing or discontinuing antimetabolites to enhance vaccine responsiveness without improving the risk of acute rejection or graft loss.^25^ However, such decisions must be made cautiously and supported by further evidence. An ongoing prospective open-label trial is evaluating the impact of immunosuppression adjustment on COVID-19 Vaccination Response in KTRs (ADIVKT; NCT05060991).

Humoral and cellular immune responses were overall comparable between patients’ receiving mycophenolate or azathioprine, with a trend toward lower cellular detection in the azathioprine group. Also, COVID-19 incidence and death were similar in both groups, although earlier in the MPS group, maybe because of the limited number of patients in the study. The transplant outcomes, such as delayed graft function and CMV infection were expected to be more frequent in the MPS arm, given donor profile and the institutional immunosuppression protocol.

Some limitations should be acknowledged. This was a single-center study, with a low number of patients, which may limit the generalizability of our findings to broader KTR populations with different demographic and clinical characteristics. Nevertheless, although the study may be underpowered to detect subtle differences, it still provides valuable real-world data from a unique kidney transplant cohort. This study included only the ChAdOx1 vaccine, and the results may not be directly extrapolated to other vaccine platforms, such as mRNA-based or inactivated vaccines. Since this study was performed during the COVID-19 pandemic, we could not direct that KTRs stop receiving additional vaccine doses. Finally, the observational design precludes definitive conclusions about causality between immunosuppressive regimens and vaccine response.

In summary, this study sheds light on the antibody kinetics and neutralizing activity in KTRs receiving different immunosuppressive regimens after ChAdOx1 primary vaccination. While azathioprine appears to confer a transient advantage in early humoral responses, the overall vaccine-induced immunity in both groups benefited substantially from booster doses. These findings underscore the importance of continued vaccine prioritization and tailored strategies to protect this high-risk population during the COVID-19 pandemic.

## Authors' contributions

Research design: HTS, LRM, JMP and RDF; Performance of the research: JSR and RMS; Writing of the paper: JSR, RMS and RDF; Data analysis: HTS, LRM, JMP and RDF.

## Funding

This study was financed by the São Paulo Research Foundation (FAPESP), Brazil. Process Number #2021/13,680–6.

## Data availability statement

The datasets generated during this study are available from the corresponding author upon reasonable request.

## Conflicts of interest

The authors declare no conflicts of interest.
